# MicroRNA-206 down-regulated human umbilical cord mesenchymal stem cells alleviate cognitive decline in D-galactose-induced aging mice

**DOI:** 10.1038/s41420-022-01097-z

**Published:** 2022-07-04

**Authors:** Yuying Zhang, Weiyue Deng, Wei Wang, Aishi Song, Omar Mukama, Sihao Deng, Xiaobo Han, Jean De Dieu Habimana, Kexin Peng, Bin Ni, Shusheng Zhang, Jufang Huang, Xiao-xin Yan, Zhiyuan Li

**Affiliations:** 1 Department of Anatomy and Neurobiology, Xiangya School of Medicine, Central South University, Changsha, China; 2 CAS Key Laboratory of Regenerative Biology, Guangdong Provincial Key Laboratory of Stem Cell and Regenerative Medicine, Guangzhou Institutes of Biomedicine and Health, Chinese Academy of Sciences, Guangzhou, China; 3 NHC Key Laboratory of Birth Defect for Research and Prevention, Hunan Provincial Maternal and Child Health Care Hospital, Changsha, China; 4 Changsha Stomatological Hospital, Changsha, China; 5 Bioland Laboratory, Guangzhou, China; 6 GZMU-GIBH Joint School of Life Sciences, Guangzhou Medical University, Guangzhou, China; 7 GIBH-HKU Guangdong-Hong Kong Stem Cell and Regenerative Medicine Research Centre, GIBH-CUHK Joint Research Laboratory on Stem Cell and Regenerative Medicine, Guangzhou, China

**Keywords:** Cognitive ageing, Ageing

## Abstract

**Background:**

Non-pathological cognitive decline is a neurodegenerative condition associated with brain aging owing to epigenetic changes, telomere shortening, stem cells exhaustion, or altered differentiation. Human umbilical cord mesenchymal stem cells (hUCMSCs) have shown excellent therapeutic prospects on the hallmarks of aging. In this study, we aimed to elucidate the role of hUCMSCs with down-regulated miRNA-206 (hUCMSCs anti-miR-206) on cognitive decline and the underlying mechanism.

**Methods:**

After daily subcutaneous injection of D-gal (500 mg/kg/d) for 8 weeks, 17-week-old male C57BL/6 J mice were stem cells transplanted by lateral ventricular localization injection. During the 10-day rest period, were tested the behavioral experiments applied to cognitive behavior in the hippocampus. And then, the mice were sacrificed for sampling to complete the molecular and morphological experiments.

**Results:**

Our behavioral experiments of open field test (OFT), new object recognition test (NOR), and Y-maze revealed that D-galactose (D-gal)-induced aging mice treated with hUCMSCs anti-miR-206 had no obvious spontaneous activity disorder and had recovery in learning and spatial memory ability compared with the PBS-treated group. The hUCMSCs anti-miR-206 reconstituted neuronal physiological function in the hippocampal regions of the aging mice with an increase of Nissl bodies and the overexpression of Egr-1, BDNF, and PSD-95.

**Conclusion:**

This study first reports that hUCMSCs anti-miR-206 could provide a novel stem cell-based antiaging therapeutic approach.

## Introduction

Aging is a gradual decline of the biological functions of multiple organs in the body, among which the brain is one of the most susceptible [1, 2]. The nonpathological changes increase in the process of brain aging, characterized by a cognitive decline, manifesting in the decline of learning, memory, and recognition ability [3]. Although the neural aging mechanism remains elusive, the hippocampus, which performs the learning and memory functions, is greatly involved [4]. The hippocampus has a huge network of intermediate neurons. The main neurons in these regions are granulosa cells in the DG region and pyramidal cells in CA1 and CA3 of the Ammon’s horn, which forms a unidirectional tri-Synaptic pathway. This network unit has been shown to affect cognition, which in turn affects memory and mood [5]. The hippocampus neurodegeneration is associated with a decrease in cellular transcription level, neuronal physiological function, synaptic plasticity [6, 7], and cognitive function [8–12]. Brain aging can be induced with D-gal, which causes oxidative stress-mediated superoxide anion free radicals. Studies have shown that long-term subcutaneous injection of high-dose D-gal in rodents resulted in cell swelling, metabolic disorders, and accelerated aging processes [13].

MSCs’ unique propensity to proliferate and differentiate has been exploited in tissue/organ repair and transplantation such as heart, bone and lung [14–16]. However, transplanted MSCs have shown low differentiation efficiency and survival rate decrease in the host [17] due to paracrine substances that contain a lot of cellular regulatory factors, and overexpressed exosomal microRNAs [18]. For instance, stem cell therapy paracrine function has been improved through in vivo microenvironment control, immune regulation, inhibition of apoptosis, and nutritional support [19, 20]. Among differentially expressed microRNAs, miR-206 targets brain-derived neurotrophic factor (BNDF), an important member of the neurotrophic factor family, mainly active in gray matter regions such as the cortex, hippocampus, and basal forebrain that protect the nervous system. Zhang et al. reported that MSCs with lower miR-206 showed improved survival in the infarcted heart [21]. Besides, J. Liu et al. showed that miR-206 overexpression reversed MSCs-Exo-mediated attenuation of chondrocyte injury [22].

Here, we elucidated that hUCMSCs with lower miR-206 improved neuroprotection and cognition in a D-gal-induced aging mouse model, and behavioral testing demonstrated downstream mechanisms through the expression of Egr-1, BDNF and PSD-95 neurotrophic factors in the hippocampus. Overall, this study provides new insight into stem cell anti-aging therapeutic.

## Results

### Characterization of hUCMSCs

**Fig. 1:**
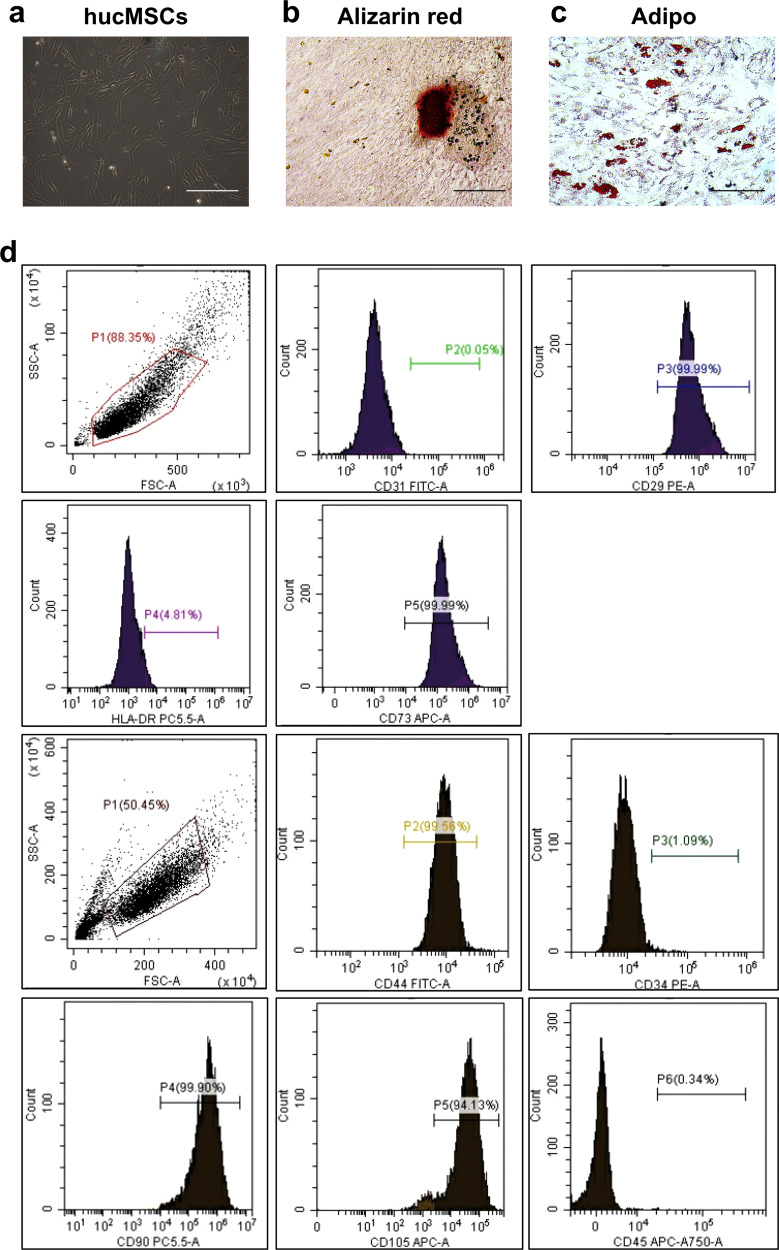
hUCMSCs morphological characterization and expression profile. **a** Spindle-shaped morphology of hUCMSCs (bar = 50 µm). **b** Osteogene-derived hUCMSCs (bar = 200 µm). **c** adipose-derived hUCMSCs (bar = 50 µm). **d** Fluorescence activated cell sorting (FACS) analysis of hUCMSCs with positive CD44, CD90, CD105, CD29, CD73, and negative CD34, CD45, CD31 and HLA-DR (*n* = 6, *p* < 0.05).

hUCMSCs from human umbilical cord can proliferate in culture plates (Fig. 1a). In vitro differentiation experiment showed that hUCMSCs successfully differentiated into osteoblasts or adipocytes under the special induction medium (Fig. 1b, c). Flow cytometry marker identification provided corroborating results, that hUCMSCs showed positive for CD29 (99.87%), CD44 (99.31%), CD73 (94.41%), CD90 (99.17%) and CD105 (99.17%), and negative for CD34 (1.64%), CD45 (0.22%), CD31 (0.23%) and HLA-DR (0%), confirming the presence of human stem cells (Fig. 1d).

### hUCMSCs improved the learning and spatial memory ability of mice

Long-term subcutaneous injection of high-dose D-gal increases free radical-mediated oxidative stress in mice, causing premature brain aging, hippocampus damage, and eventually cognitive decline [13]. The effect of D-gal on cognitive behavior was analyzed using open-field activity (OFT), a new object recognition test (NOR) and Y-maze) in the hippocampus during a 10-day rest period after treatment of the 18-week-old male C57BL/6 J mice. The mice were randomly divided into four groups: normal, D-gal treated with PBS (D-gal+PBS), hucMSCs (Cell), and miR-206 down-regulated hucMSCs (Cell-anti-miR-206). After daily subcutaneous injection of D-gal (500 mg/kg/d) for 8 weeks, mice were injected in the lateral ventricle with no-treated hucMSCs or pre-treated with Cell-anti-miR-206, separately. During the 10-day rest period, the OFT, NOR, and Y-maze were completed. Mice were sacrificed on the 10th day to remove the brain hippocampus for molecular and morphological subsequent experiments.

**Fig. 2:**
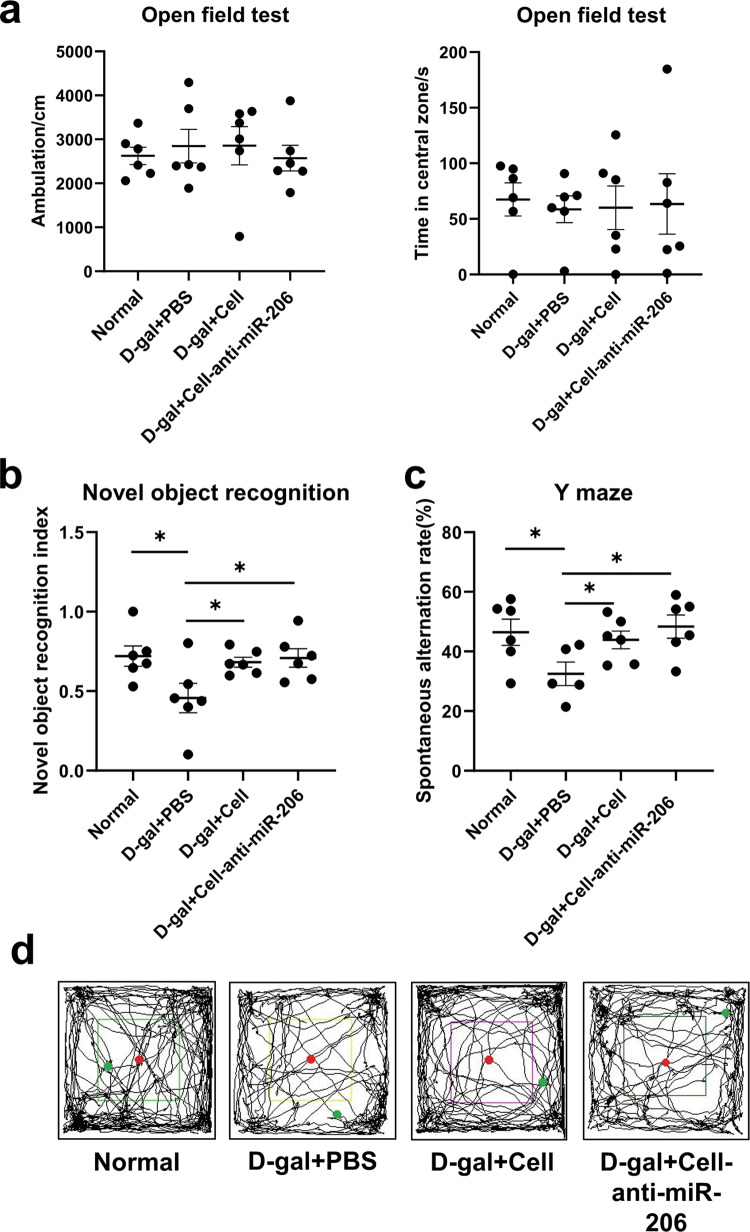
Learning and spatial memory were restored in mice after stem cell transplantation. **a** OFT experimentation of spontaneous movement ability and anxiety of mice. **b** The learning and memory ability of mice tested by NOR. **c** The spatial memory ability of mice tested by Y-maze. **d** Spontaneous movement of mice in an open field trajectory diagram. The results were expressed as the Mean ± SD, *p < 0.05, versus D-gal+PBS groups, *n* = 6.

The OFT was performed to test the motor ability of mice. As shown in Fig. 2a, d, the results were not statistically different, indicating no obvious spontaneous activity disorder and anxiety. The NOR test was performed to observe the short-term learning and memory ability of mice. Compared with the normal group, the new object recognition index (NOI) of the D-gal group decreased and NOI increased significantly after cell transplantation (Fig. 2b). This showed that D-gal-induced aging mice had decreased learning memory, which recovered after cell intervention.

The Y-maze was performed to observe the short-term spatial working memory of mice. The results showed that the spontaneous alternation rate of D-gal mice decreased, and after cell transplantation increased compared with the PBS control group (Fig. 2c). It is noteworthy to mention that although there was no significant difference in OFT between the cell and the cell-anti-miRNA206 groups; a difference was observed in NOR and more significant in Y-maze (Fig. 2b, c). Overall, the huMSCs improved the learning and spatial memory ability of mice.

### hUCMSCs intervention through a paracrine mechanism

**Fig. 3:**
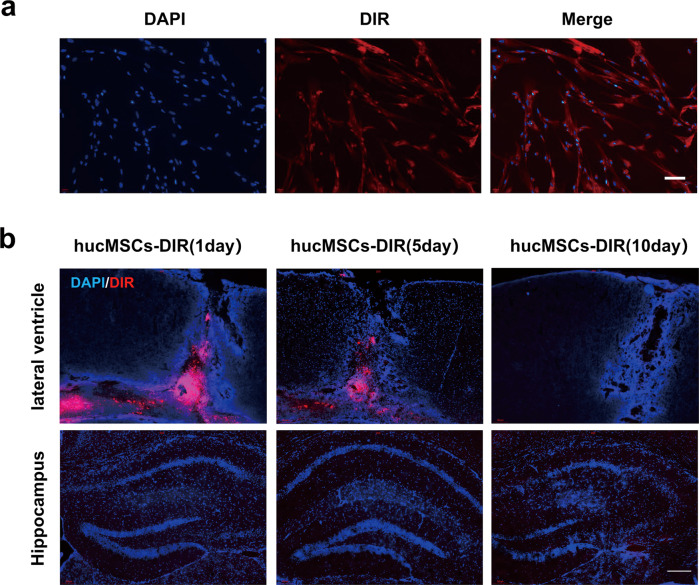
hUCMSCs-DIR detection in mice brains. **a** Detection of hUCMSCs-DiR and cell nucleus stained with DAPI in the lateral ventricle. **b** Detection of huCMSCs-DIR in the hippocampus, Bar = 50 µm, *n* = 6.

To track hUCMSCs, we stained hUCMSCs with DIR dye before transplantation. Immunofluorescence staining showed highly stained cells (Fig. 3a). After lateral ventricle injection transplantation, cells were monitored for 1, 5, and 10 days. The signal decreased gradually in time-dependent and there was no detectable trace of hUCMSCs in the hippocampus after 10 days (Fig. 3b), suggesting hUCMSCs migration through paracrine exosomes to stimulate treatment.

### BDNF is negatively regulated with miR-206

**Fig. 4:**
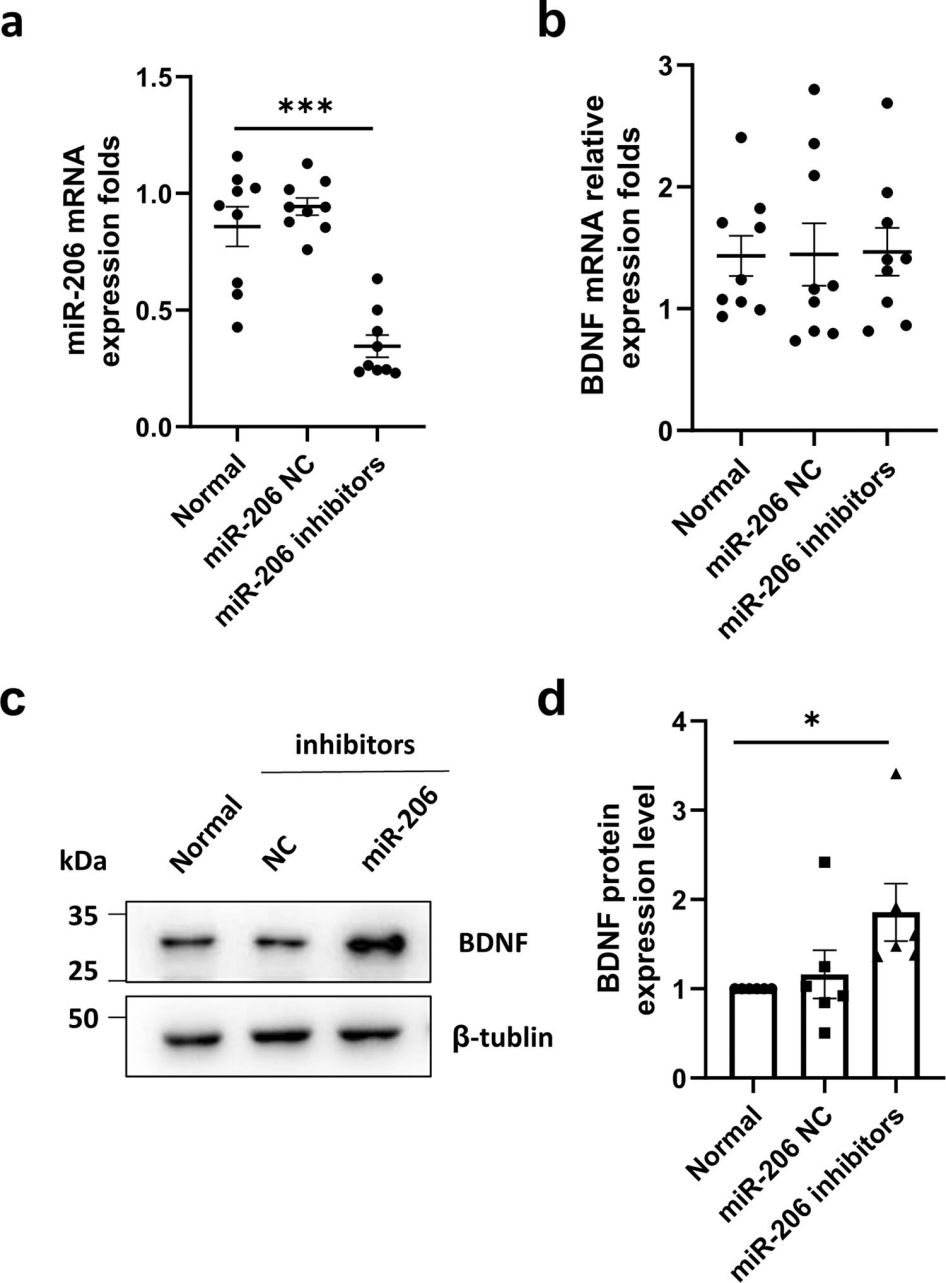
Relationship between miR-206 and BDNF. **a** After the transfection of hUCMSCs with miR-206 inhibitor, the expression level of miR-206 mRNA was detected by RT-qPCR. **b** After transfection with miR-206 inhibitor, the expression level of BDNF mRNA was detected by RT-qPCR. **c** After transfection of hUCMSCs with miR-206 inhibitor, the expression level of BDNF protein was detected by western blot. **d** Quantitative analysis of the protein levels of BDNF. The results were expressed as the Mean ± SD, *p < 0.05, versus normal groups, *n* = 6.

MicroRNA, as a post-transcriptional regulatory element, is involved in the post-transcriptional regulation of target gene expression [23]. The target Scan Human Database analysis showed that miR-206 targeted BDNF, suggesting that miR-206 may be associated with neuroprotection. To verify whether miR-206 regulates BDNF, we transfected hUCMSCs with miR-206 inhibitor and NC. RT-qPCR showed no significant difference in BDNF mRNA expression (Fig. 4b). However, western blot showed that the protein expression level of BDNF increased significantly (Fig. 4C, D). The results showed that the low expression of miR-206 promoted the high expression of BDNF in hUCMSCs, and BDNF was negatively regulated with miR-206, confirming that miR-206 could directly bind to the BDNF.

### hUCMSCs with down-regulated miR-206 achieve neuroprotective effects by targeting BDNF in vivo

**Fig. 5:**
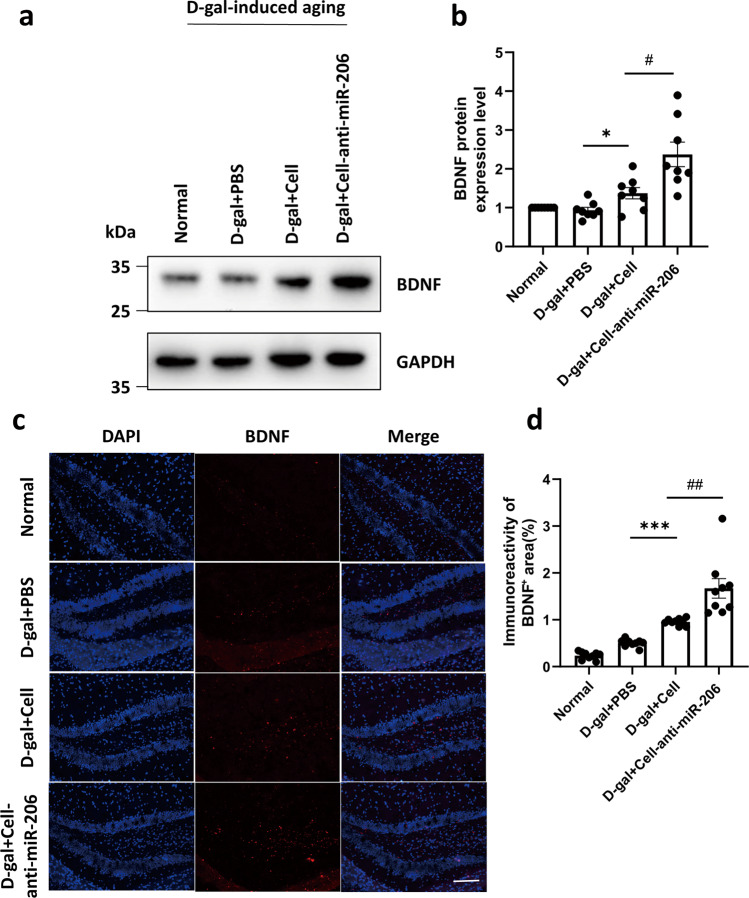
The low expression of miR-206 in hUCMSCs promoted the expression of BDNF in vivo. **a** The protein levels of BDNF were determined by western blotting. **b** Quantitative analysis of the protein levels of BDNF using ImageJ. **c** Representative micrographs for BDNF immunofluorescence staining of hippocampus from mice in four groups (*n* = 5 per group). **d** Quantification of BDNF-positive cells in four groups. The results were expressed as the Mean ± SD, *p < 0.05, n.s., nonsignificant, bar =50 µm, *n* = 6.

The above results confirmed that BDNF is negatively regulated with miR-206. In addition, to study the effect of low expression of miR-206 on brain aging, we performed immunofluorescence staining of anti-BDNF antibody and western blot in the hippocampus of mice. As shown in Fig. 5, the results showed that the low expression of miR-206 in hUCMSCs promoted the expression of BDNF in the damaged hippocampus. These results revealed that miR-206 downregulated hUCMSCs exhibited neuroprotection.

### hUCMSCs with downregulated miR-206 promoted the physiological function of hippocampal neurons

**Fig. 6:**
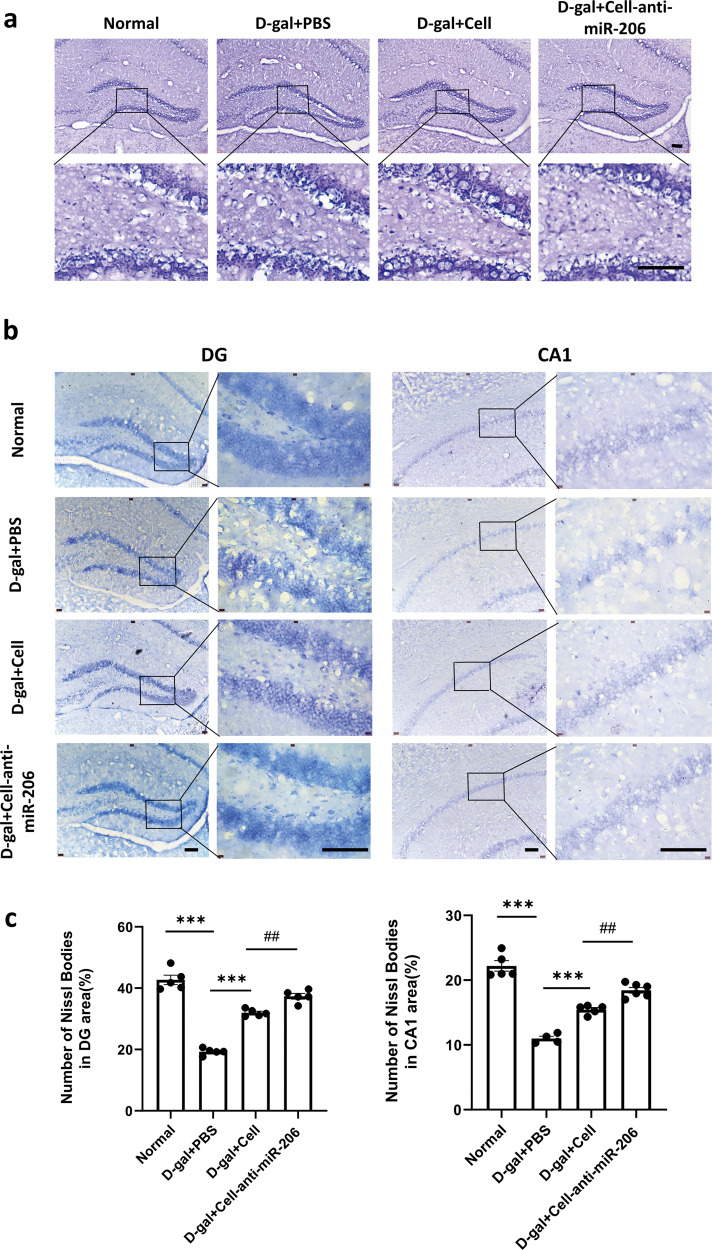
The low expression of miR-206 of hUCMSCs significantly improved the physiological function of nerve cells in the hippocampus. **a** HE staining of the hippocampus of D-gal-induced aging mice. **b** Nissl staining of the hippocampal CA1 and DG regions of D-gal-induced aging mice. Bar = 200 µm. Enlarged pictures after the black frame. Bar = 50 µm. **c** Quantitative analysis of the number of neurons using ImageJ. The results are expressed as the Mean ± SD, *p < 0.05, verses D-gal+PBS groups, ^#^*p* < 0.05, verses D-gal+Cell groups (*n* = 6).

Ten days after hUCMSCs transplantation, mouse brain coronal sections were HE stained, which showed no mass cell shrinkage in the hippocampus of each group, and the cell structure was complete and orderly (Fig. 6a). The results showed that there was no large number of neuronal apoptosis, which was consistent with non-pathological aging. However, more differences were observed in the Nissl staining. Nissl staining was performed to observe the changes in hippocampal CA1 and DG regions, involved in memory formation. The Nissl body staining was deeper, and neuron morphology was regular. In the D-gal+PBS group, the number of Nissl bodies decreased, the staining was shallow, and the morphology of neuron cells was irregular. After transplantation of hUCMSCs with lower miR-206, the number of Nissl bodies increased, and the staining was deeper than that of the D-gal +Cell group (Fig. 6b, c). The Nissl bodies’ increase can be attributed to the reconstitution of protein synthesis in neurons and their normal physiological functioning, suggesting that hUCMSCs with lower miR-206 promoted the physiological function of hippocampal neurons.

### HUCMSCs with down-regulated miR-206 promoted the expression of learning and memory-related proteins

**Fig. 7:**
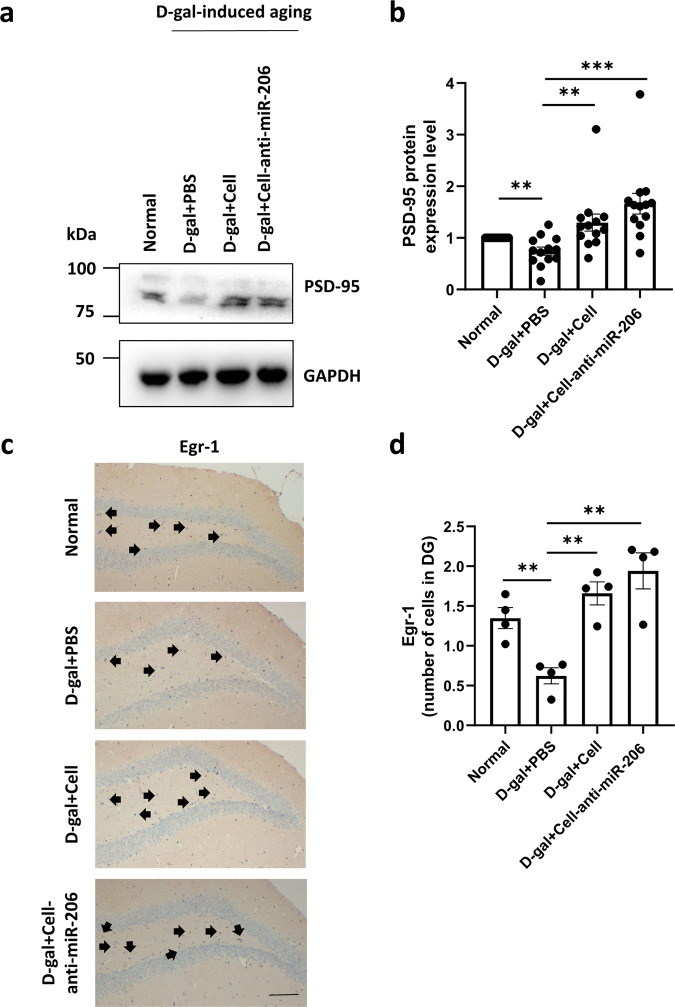
The low expression of miR-206 of hUCMSCs promoted the expression of PSD-95 and Egr-1. **a** The protein levels of PSD-95 were determined by western blotting. **b** Quantitative analysis of the protein levels of PSD-95 and using ImageJ. **c** Egr-1 in the hippocampus was detected by immunohistochemistry, Bar = 50 µm. **d** Quantitative analysis of the protein levels of Egr-1 by ImageJ. The results were expressed as the Mean ± SD, *p < 0.05, versus D-gal+PBS groups (*n* = 6).

Postsynaptic density-95 (PSD-95) is the main component of the excitatory postsynaptic membrane is dense and plays an important role in synaptic signaling, learning, and memory. Studies have shown that PSD-95 can promote synaptic maturation and enhance synaptic plasticity [24]. Therefore, PSD-95 protein expression level was detected, which was significantly upregulated in tissue samples of an anti-miR-206 group (Fig. 7a, b). We then detected Egr-1, an immediate early gene and one of the important molecules involved in the formation and consolidation of long-term memory of synaptic plasticity of neurons [25], and its decrease impairs spatial learning and memory ability [26]. Intriguingly, our immunohistochemical staining results showed Egr-1 expression upregulated in the dentate gyrus in the anti-miR-206 group (Fig. 7c, d).

## Discussion

Aging is an inevitable process. With the aggravation of population aging, minimizing or alleviating aging is imperative to potentially slow down age and age-related diseases [27]. Stem cell- therapy promises anti-aging owing to its rapid and efficient therapeutic effect, precision delivery, and safety.

MicroRNAs are a class of endogenous small non-coding RNAs responsible for post-transcriptional regulation of gene expression. Recent studies showed that miR-206 targets BDNF, which regulates anxiety-related behaviors and neuropathic pain induced by chronic contraction injury [28, 29]. The miR-206-knockdown exosomes therapy was more effective in reducing neuronal death and improving neurobehavioral scores during early brain injury (EBI) [30]. We hypothesize that microRNAs play a key role in communication between MSCs and parenchymal cells. In this study, we focused on the relationship between miR-206 abundance in hUCMSCs paracrine and neuroprotection. By decreasing the expression of miR-206 of hUCMSCs, cells were transplanted into D-gal-induced mouse aging. Our results showed that the BDNF of hUCMSCs was upregulated after the down-regulation of miR-206. Due to microRNA being responsible for the post-transcriptional regulation of gene expression, we detected no difference in the mRNA level of BDNF, but the protein expression was upregulated. Therefore, we know that miR-206 targets BDNF and is negatively regulated. In addition, we detected that after down-regulation of miR-206, the expression of BDNF in the hippocampus of mice was significantly increased. And Nissl staining showed that the state of neurons in the DG and CA1 regions of the hippocampus of mice was restored to varying degrees after stem cell intervention. Nissl staining can reflect the generative and synthetic ability of nerve cells, which indicates that the physiological state of nerve cells in the hippocampus was better improved. Suggesting that miR-206 may be a key regulator of intervention in D-gal-induced aging.

Recently, proteins containing the PDZ motif have been proposed as a molecular scaffold of receptors and synaptic cytoskeletal elements. The prototypical PDZ protein, PSD-95/SAP-90, is a membrane-associated guanylate kinase (MAGUK) concentrated at a glutamatergic synapse. PSD-95 is the main component of excitatory postsynapses, which plays an important role in the formation and maturation of synapses and synaptic signaling, learning, and memory functions. Meanwhile, D-gal as a common model of aging research, induced senescence has been mostly used in pharmacologic studies. Our results also showed that after D-gal induced senescence, the protein expression level of PSD-95 in the hippocampal tissues of mice decreased significantly, which was recovered after stem cell transplantation. Egr-1 is also involved in learning and memory and synaptic plasticity, memory formation, and consolidation. We detected that the expression of Egr-1 was down-regulated after D-gal induced senescence, then up-regulated after treated hUCMSCs with lower miR-206. Therefore, we think that hUCMSCs with down-regulated miR-206 have a better neuroprotective effect by targeting BDNF in D-gal-induced aging mice.

Upstream CREB regulates Egr-1. When p-CREB, the activated form of CREB, is continuously enhanced in the hippocampus, the expression of downstream Egr-1 will be promoted, thus affecting learning and memory ability [31]. CREB is a key transcription factor in the activation signal of TrkB, which is the binding receptor of BDNF. Upregulation of BDNF/TrkB/CREB this classic neuroprotective pathway signaling inhibits neuronal apoptosis in neurodegenerative diseases [32]. Therefore, we consider that miR-206 may improve aging cognition through the BDNF/TrkB/CREB signaling pathway by targeting BDNF in D-gal-induced aging mice.

In our study, the tracing of stem cells in the brain showed no cells in the lateral ventricle after 10 days. We also tested three short-term memory behavioral experiments related to episodic and spatial learning. However, it is also necessary to explore long-term learning and memory ability of cognitive ability, which can be explored by other related behavioral tests in future studies. In addition, we showed that miR-206 targets BDNF to improve cognitive decline by exploring key intermediate molecules associated with the signaling pathway, suggesting further studies on the regulation mechanism of its internal signal pathway.

## Conclusion

Our results first report that miR-206 targets BDNF and negatively regulates BDNF expression. hUCMSCs with down-regulated miR-206 can improve the physiological function of nerve cells in the hippocampus and promote the expression of PSD-95 and Egr-1 related to learning and memory. The data propose that the neuroprotective role of hUCMSCs with down-regulated miR-206 is one strategy to improve the cognitive decline of aging.

## Methods

### Animals

In this experiment, a total of 60 C57BL/6 J mice (Hunan Slack Jingda Experimental Animal Co, Ltd, Changsha, China) weighing 25–30 g at 9 weeks old in this experiment were used. The animal model was established for eight weeks, to ensure the physical health of the animals, we selected 9-week-old in youth mice. Mice were raised in the Department of Laboratory Animal Science (Central South University, Changsha, Hunan, China) and housed individually in separate cages (SPF + IVC) and had free access to food and water and 12 h light-dark cycle for seven days before the experiment. All animal experiments were according to the “Guide for the Care and Use of Laboratory Animals, 8th ed., 2010” (National Institutes of Health, Bethesda, MD) and were approved by the Institutional Animal Care and Use Committee of Central South University (Changsha, China; Permit Number: 2020sydw0913).

### hUCMSCs Culture

hUCMSCs were from the HYS Cell Gene Engineering company (Changsha, Hunan, China). The hUCMSCs were cultured using F12-DMEM (DMEM, Gibco, Grand Island, NY, USA), which contained 10% FBS (FBS, Gibco, Grand Island, NY, USA) for the primary culture (37°C, 5% CO_2_), and then passaged after reaching 80-90% confluence. P5 hUCMSCs were used in all experiments and counted using a hemocytometer. Cells phenotypes were analyzed by flow cytometry. The P5 hUCMSCs were characterized using CD34, CD44, CD45, CD29, CD31, CD73, CD90, CD105 and HLA-DR antibodies (Biolegend, Way San Diego, CA, USA). When hUCMSCs reached 80-90% confluence, hUCMSCs were seeded in a six-well plate with approximately 5 × 10^6^ cells per well. After 24 h, Mesenchymal Stem Cell Osteogenic Differentiation Kit (5011-024-K, Trevigen, MD, USA) was used to induce osteogenic differentiation of hUCMSCs. Calcium nodules formed after osteogenic induction were stained with Alizarin Red. The hUCMSCs were induced with a mesenchymal stem cell adipogenic differentiation kit (5010-024-K, Trevigen, MD, USA) to induce adipogenic differentiation and stained with oil red O.

### Cell transfection

The miR-206 inhibitors and negative control (NC) (GenePharma, Shanghai, China) were transfected into hUCMSCs at a final concentration of 50 nmol/L. After recovering, P5 hUCMSCs were cultured into the 6-well plates until their density reached 80–90%. The miR-206 inhibitors or NC and Lipofectamine 3000 Transfection Reagent (L3000001, Thermo Fisher Scientific, MA, USA) were diluted with Opti-MEM (31985062, Gibco, Grand Island, NY, USA) and cells were cultivated with Opti-MEM. After 6 hours of transfection, the hUCMSCs were transferred to DMEM medium supplemented with 10% FBS for 48 h prior to post-transfection efficacy measuring by RT-PCR or Western blot.

### Animal model experimentation and cell transplantation

D-gal (500 mg/kg/d) was subcutaneously injected into the back of each C57 mouse once a day for 8 weeks. The normal group injected the same volume of saline. Mice were anesthetized with an intraperitoneal injection of pentobarbital (ZaoZhuang, Shandong, China) at about 10 mg/kg. According to the stereotaxic map of a mouse brain, the anterior fontanelle is backward 2.0 mm, the sagittal suture (mediolateral, ML) was 1.5 mm left and right, and the insertion depth (dorsoventral, DV) was 2.5 mm. 10 µl PBS or MSCs cell suspension was slowly injected into the lateral ventricle with microliter syringes and stopped for 2 min after each injection of 2 µl. After completion, the syringe was stopped for 5 min.

The mice were randomly assigned to 4 groups: normal (*n* = 15) control group, D-gal+PBS (*n* = 15), D-gal+Cell (*n* = 15), D-gal+Cell-anti-miR-206 (*n* = 15).

After the operation, at 10 days after injection of MSCs, all the mice were sacrificed and taken out the hippocampus and whole brain.

### Tracers after cell transplantation

P5 hUCMSCs were collected and plated into the 6-well plates or circle microscope cover glass. When it reaches 80–90%, cells were stained with DIR Iodide (DiIC18) (Maokangbio, Shanghai, China) at 37 °C for 20 min according to the protocol of the manufacture (7). The cells were resuscitated, washed, and collected using PBS. Then, the glass slide was DAPI stained for nuclear observation under fluorescence microscopy. The collected cells were injected into the lateral ventricle of the mouse brain, and the after OCT embedded brain was cut into serial 15-µm-thick coronal sections and DAPI stained for fluorescence microscopy.

### Behavioral experiment

The open-field test (OFT): A 40 × 40 × 40 cm cube box was used. The bottom of the box was divided into 25 squares of equal size, from which nine squares in the center were defined as the central region and the rest as the peripheral region. Each mouse, in turn, was placed in the central area and left to explore for 10 min [33].

The new object recognition test (NOR): it is divided into three periods: adaptation, training and testing. The first day was the adaptation period. The mice were placed in the middle of the open field in turn and explored freely for 5 min. Twenty-four hours later, entering the training period, two identical objects were placed in the open field with an interval of 20 cm or 10 cm between the two objects and the sidewall. Each mouse was placed from the central line attached to the wall on the side of the non-object area of the open field and was left to explore for 5 min. After a rest of 1 h, entering the testing period, one of the objects in the open field was removed and replaced with a new object with the same material but different colors and sizes. Then, the mice were placed in the same position in the box in turn and left to explore freely for 5 min. Then calculate according to the formula: new object recognition index(NOI) = exploring new object time/(exploring new object time+ exploring old object time) ×100% [34, 35].

The Y-maze: three identical 30 × 5 × 15 cm gray closed arms with a 120° angle between the three arms(new arm, different arm and starting arm), and a triangle middle joint part. There are two periods: the training period and the testing period. In the training period, the new arm was closed, and the mice were placed in the starting arm facing the middle joint part in turn and left to explore for 10 minutes. After a rest of 1 h, open the new arm, and the mice were tested for free exploration for 5 min in turn. When the mouse limbs completely entered the arm, it was judged as arm entry. When the third arm entry was different from the first two, it was considered the correct arm entry. Then calculate according to the formula: spontaneous alternation rate% = (Number of Alternations/[Total number of arm entries-2]) ×100% [36].

In all three behavioral experiments, the mice were placed in a behavioral chamber an hour earlier to acclimate to their environment. Before the experiment, including after each mouse finished the test, the apparatuses were cleaned and wiped with 70% alcohol and keep them dry, eliminating the interference of biases and olfactory cues. Each mouse completed the test and was placed in a separate cage to avoid interaction with the untested mice.

The hippocampal-involved behavioral experiments were completed in the Department of Laboratory Animal Science (Central South University, Changsha, China). An infrared camera was installed on the top of the site and connected with a computer to track the mouse. The experimental data was collected and analyzed by the Smart3.0 software system.

### RNA isolation and RT-qPCR

Total RNA was extracted from cells using Trizol (Thermo, USA) according to the manufacture protocol. RNA was reverse transcribed into cDNA using a cDNA synthesis kit (Cwbio, Beijing, China). Subsequently, the cDNA product was used as a template for PCR using SYBR green mixture (Cwbio, Beijing, China). The tests were performed in triplicate, and data were analyzed using the 2^-ΔΔCt^ method. U6 and Actin were used as internal controls. The U6, Actin, and BDNF primers were designed as shown in the following table.

**Table:** 

Primer name	Primer sequence (5’-3’)
Actin	ACCCTGAAGTACCCCATCGAG (F)
	AGCACAGCCTGGATAGCAAC (R)
BDNF	GGCTTGACATCATTGGCTGAC (F)
	CATTGGGCCGAACTTTCTGGT (R)
U6	CTCGCTTCGGCAGCACA (F)
	AACGCTTCACGAATTTGCGT (R)
hsa-miR-206	GGTGGAATGTAAGGAAGTGTGTGG

### Western blot analysis

Total protein was extracted from cultured cells or tissue using RIPA (CWBIO, Shanghai, China) and protease inhibitor (CWBIO, Shanghai, China). Protein concentration was determined using a BCA Protein Assay Kit (Thermo Scientific, Rockford, IL, USA). Equal amounts of proteins were electrophoresed in 10% SDS-PAGE and electro-transferred onto polyvinylidene difluoride (PVDF) membrane (Millipore, Billerica, MA, USA), and sealed with 5% skimmed milk for 2 h at room temperature and incubated with anti-BDNF antibody(1:1000, Abcam, USA) or anti-PSD95 antibody(1:1000, Proteintech, Wuhan China) overnight at 4 °C. After being washed, the membranes were incubated with horseradish peroxidase (HRP)-conjugated secondary antibodies for 1 h at room temperature. Protein bands were detected by an enhanced chemiluminescence kit (Thermo Scientific, Rockford, IL, USA). Image J software (National Institutes of Health, USA) was used to quantify the band densities.

### Morphology staining

After mice-intracranial perfuse with PBS followed by 4% paraformaldehyde (PFA), the whole brain was fixed with PFA solution (Sinopharm Chemical Reagent Co. Ltd, Shanghai, China) i.e. 4% PFA in 0.1 M PBS (Solarbio, Beijing, China) at 4 °C for 24 h. Then, it was dehydrated with 15% sucrose solution at 4 °C for 24 h and replaced with 30% sucrose solution for 24 h the next day. Brains were then cut into serial 15-µm-thick coronal sections (Leica, Wetzlar, Germany) and mounted onto glass slides. Tissue sections were stained with Hematoxylin-Eosin (HE) (Beyotime, Shanghai, China) or with Nissl body staining Kit (Meilun, Dalian, China) and finally studied using light microscopy. Images were captured on Nikon confocal microscope (Nikon Instruments, Inc., Japan).

### Immunohistochemistry and immunofluorescence

For immunohistochemistry staining, tissue sections were incubated in 0.3% hydrogen peroxide/PBST (Solarbio, Beijing, China) for 30 min. The sections were then sealed with normal horse serum (Beyotime, Shanghai, China) /PBST (1:200) for 2 h. Then, they were incubated with anti-Egr1 antibody(1:100, PTG, USA) overnight at 4 °C, washed with PBS, and then incubated with HRP labeled Goat Anti-Rabbit IgG antibody(1:500, ZSGB-BIO, Beijing China) at 37 °C for 2 h. At last, the slices were washed with PBS and sealed with the coverslip by neutral resin (Sinopharm Chemical Reagent Co. Ltd, Shanghai, China).

For immunofluorescence staining, the sections were blocked in normal donkey serum/PBST (1:200) for 2 h. Then, they were incubated with anti-BDNF antibody (1:1000, Abcam, USA) at 37 °C for 1 h and washed with PBS. Subsequently, the sections were incubated with secondary antibodies (Alexa Fluor Cy5, 1:800; APExBIO, USA). Cell nuclei were stained with DAPI (1:1000, DAPI, Sigma Aldrich, MO, USA) and images were captured on Nikon confocal microscope (Nikon Instruments, Inc., Japan). At last, the slices were sealed with the coverslip by glycerin (Solarbio, Beijing, China).

### Statistical Analysis

The data are expressed as Mean ± SD. The software Prism Graph Pad (version 7.0, La Jolla, CA) was used to perform the statistical analysis. One-way ANOVA and Turkey’s test (analysis of variance) were used to verify the differences between groups. *p* < 0.05 was considered statistically significant.

## Supplementary information

Original Western Blots Data file

Reproducibility checklist

## Data Availability

All relevant data and materials are available from the authors upon reasonable request.
